# Commentary: Reciprocal Modulation of I_K1_–I_Na_ Extends Excitability in Cardiac Ventricular Cells

**DOI:** 10.3389/fphys.2016.00647

**Published:** 2016-12-23

**Authors:** Birgit Goversen, Teun P. de Boer, Marcel A. G. van der Heyden

**Affiliations:** Division of Heart and Lungs, Department of Medical Physiology, University Medical Center UtrechtUtrecht, Netherlands

**Keywords:** reciprocal modulation, inward rectifier current, sodium current, induced pluripotent stem cells, arrhythmia, K_IR_2.1, Na_v_1.5

We read with great interest the excellent paper by Varghese (2016) who describes an *in silico* approach to study the consequences of reciprocal regulation of expression of the sodium current (I_Na_) and the inward rectifier potassium current (I_K1_) in the ventricle for cardiac excitability and conduction. The *in silico* approach follows the experimental results obtained by Milstein et al. (2012) who demonstrated functional co-regulation of the sodium and inward rectifier currents, and their underlying channel proteins Na_v_1.5 and K_IR_2.1 respectively, and electrophysiological consequences upon overexpression in rodent cardiomyocytes with respect to action potential duration and re-entry based arrhythmia propensity. Varghese adapted the guinea pig ventricular cardiomyocyte model, developed by Noble et al. (1998), that is extrapolated to simulations for one dimensional cardiac fibers, in which the fiber is represented as a linear cable model. Varghese changed either the conductance for I_Na_ and I_K1_ individually or in tandem, to assess their influence on the excitability of mammalian ventricular cardiomyocytes. One of the most interesting findings in this paper is the dominance of I_K1_ over I_Na_ in regulation of cardiac excitability, which yields important questions about the significance of the inward rectifier in this process (Varghese, 2016). This commentary will put these results in a broader context in order to provide a framework for future research questions.

The experimental data of Milstein et al. (2012) point to the existence of a macromolecular complex in which the SAP97 protein may have a major role in reciprocal regulation of expression of Na_v_1.5 and K_IR_2.1 proteins, since both channels present binding motifs for SAP97. Milstein and colleagues stress that the cell biological principles underlying reciprocal expression at the sarcolemma are only partly resolved and propose a role for ion channel trafficking in the process. Indeed, Na_v_1.5 promotes K_IR_2.1 protein to be presented at the cell surface, and it decreases K_IR_2.1 internalization. Whether and to which extent K_IR_2.1 affects Na_v_1.5 protein trafficking still needs to be resolved. Furthermore, a number of additional proteins are candidate in establishing Na_v_1.5-K_IR_2.1 macromolecular complexes at the plasma membrane as well as intracellularly (Willis et al., 2015). Finally, the subcellular localization of K_IR_2.1 and Na_v_1.5, e.g., intercalated disc vs. lateral membranes, may very well depend on the nature of the macromolecular complex. When location specific complexes exist, we may predict that these respond differently to disease causing factors and thereby change anisotropy. For now, the field has to elucidate the composition of (additional) molecular complexes from native cell types and more importantly, gain knowledge on Na_v_1.5 and K_IR_2.1 stoichiometry in such complexes and determine whether variations in stoichiometry between complex types exist, and if so, decipher its significance.

In contrast to Na_v_1.5, which is predominantly present in the heart, K_IR_2.1 channels are expressed in many excitable tissue types, like skeletal and smooth muscle, neuronal cells, but also in non-excitable tissues (reviewed in De Boer et al., 2010). This sets the stage for efforts to explore potential reciprocal modulation of K_IR_2.1 and various sodium channel subtypes in non-cardiac tissues, a hypothesis already put forward in the field of epilepsy (Ambrosini et al., 2014). Not only does K_IR_2.1 protein distribution differ between tissue types, there is also variation within the heart. For example, atria and Purkinje fibers express less K_IR_2.1 channels than ventricles. This spatial variation also holds true for development and disease. Transcriptional differences have been seen in development and upregulation of K_IR_2.1 has been associated with progression of atrial fibrillation (De Boer et al., 2010). All these expressional differences likely play a role in action potential formation and propagation, and it may be clear that a complete set of *in silico* models, representing different cardiac tissue types and developmental stages may be required to fully appreciate the functional roles of reciprocal modulation. Currently, both the guinea pig ventricular cardiomyocyte model as the linear cable model do not inhabit these dynamic features and by definition cannot provide clues on anisotropy.

The findings by Varghese might be of importance to the field of induced pluripotent stem cell-derived cardiomyocytes (iPSC-CMs). iPSC-CMs are of interest for tissue engineering and *in vitro* drug screening purposes, but their electrical immaturity has to be considered carefully (Jonsson et al., 2012). Aside from an ill-developed sarcoplasmic reticulum, their main drawback is spontaneous beating activity due to a lack of I_K1_ (Jonsson et al., 2012). Restoration of K_IR_2.1 and therefore I_K1_ might electrically mature these iPSC-CMs. Since the absence of I_K1_ is closely linked to very low transcription levels of the K_IR_2.1 producing gene (*KCNJ2*) in this cell type, it appears obligatory to resolve *KCNJ2* promoter regulation first and subsequently use gene specific transcription factors or their upstream regulatory pathways to increase K_IR_2.1 mRNA expression levels. Once K_IR_2.1 protein expression level is elevated, the reciprocal modulation of sodium and inward rectifier channels might provide additional means to enhance K_IR_2.1 and thus I_K1_ in iPSC-CMs. The study by Varghese shows that the interaction between sodium channel and the inward rectifier yields functional implications that cannot be ignored.

## Author contributions

BG, TdB, and MvdH wrote the submitted commentary on an original contribution by Anthony Varghese.

## Funding

This work was financially supported by a grant from ZonMW (2015-114021501).

### Conflict of interest statement

The authors declare that the research was conducted in the absence of any commercial or financial relationships that could be construed as a potential conflict of interest.
